# Amyloid-Driven Tau Accumulation on Mitochondria Potentially Leads to Cognitive Deterioration in Alzheimer’s Disease

**DOI:** 10.3390/ijms222111950

**Published:** 2021-11-04

**Authors:** Mar Cuadrado-Tejedor, Marta Pérez-González, Rocío Alfaro-Ruiz, Sara Badesso, Diego Sucunza, María Espelosin, Susana Ursúa, Mercedes Lachen-Montes, Joaquín Fernández-Irigoyen, Enrique Santamaria, Rafael Luján, Ana García-Osta

**Affiliations:** 1 Neurosciences Program, Center for Applied Medical Research (CIMA), University of Navarra, IdiSNA, 31008 Pamplona, Spain; m.gonzalez@ucl.ac.uk (M.P.-G.); sbadesso@alumni.unav.es (S.B.); mespe@unav.es (M.E.); sursuasa@unav.es (S.U.); 2 Department of Pathology, Anatomy and Physiology, School of Medicine, University of Navarra, 31008 Pamplona, Spain; 3 Synaptic Structure Laboratory, Instituto de Investigación en Discapacidades Neurológicas (IDINE), Department Ciencias Médicas, Facultad de Medicina, Universidad Castilla-La Mancha, 02008 Albacete, Spain; Rocio.Alfaro@uclm.es (R.A.-R.); Rafael.Lujan@uclm.es (R.L.); 4 Clinical Neuroproteomics Unit, Proteomics Platform, Proteored-ISCIII, Navarrabiomed, Hospital Universitario de Navarra (HUN), Universidad Pública de Navarra (UPNA), IdiSNA, 31008 Pamplona, Spain; mercedes.lachen.montes@navarra.es (M.L.-M.); jfernani@navarra.es (J.F.-I.); esantamma@navarra.es (E.S.)

**Keywords:** Alzheimer’s disease, amyloid, hTauP301L, mitochondria

## Abstract

Despite the well-accepted role of the two main neuropathological markers (β-amyloid and tau) in the progression of Alzheimer’s disease, the interaction and specific contribution of each of them is not fully elucidated. To address this question, in the present study, an adeno-associated virus (AAV9) carrying the mutant P301L form of human *tau*, was injected into the dorsal hippocampi of APP/PS1 transgenic mice or *wild type* mice (WT). Three months after injections, memory tasks, biochemical and immunohistochemical analysis were performed. We found that the overexpression of hTauP301L accelerates memory deficits in APP/PS1 mice, but it did not affect memory function of WT mice. Likewise, biochemical assays showed that only in the case of APP/PS1-hTauP301L injected mice, an important accumulation of tau was observed in the insoluble urea fraction. Similarly, electron microscopy images revealed that numerous clusters of tau immunoparticles appear at the dendrites of APP/PS1 injected mice and not in WT animals, suggesting that the presence of amyloid is necessary to induce tau aggregation. Interestingly, these tau immunoparticles accumulate in dendritic mitochondria in the APP/PS1 mice, whereas most of mitochondria in WT injected mice remain free of tau immunoparticles. Taken together, it seems that amyloid induces tau aggregation and accumulation in the dendritic mitochondria and subsequently may alter synapse function, thus, contributing to accelerate cognitive decline in APP/PS1 mice.

## 1. Introduction

Alzheimer’s disease (AD) is characterized by the presence of two histopathological marks in the brain, senile plaques and neurofibrillary tangles (NFT), containing amyloid beta peptide 42 (Aβ_42_) and phospho-tau (p-tau) respectively. Despite continued progress and efforts made, the pathological sequence events causing AD are still unknown. In patients with mild cognitive impariment (MCI), levels of Aβ_42_ in cerebrospinal fluid (CSF) are decreased years before conversion to dementia, whereas tau and p-tau levels are later markers [1], suggesting that AD starts with Aβ pathogenesis whereas tau pathology may have a role in a later state in the disease. Accordingly, the amyloid hypothesis has been, for a very long time, the main explanation for AD pathogenesis. This hypothesis states that Aβ_42_ becomes first and acts as a key driver for inducing tau pathology and tau-mediated neurodegeneration [2,3,4]. However, against this hypothesis appears the one referred as “spatial paradox” which demonstrates that Aβ and tau pathologies initially start in different brain areas following distinct temporal sequences (review in [5]). Moreover, it is already accepted that tau pathology correlates better with neurodegeneration and cognitive impairment than Aβ pathology [6]. In line with this idea, several studies have demonstrated that amyloid can physiologically accumulate and aggregate to form amyloid deposits in the brain of cognitive normal aged-individuals [7]. Likewise, Aβ-targeted therapies, despite reducing amyloid load in the brain of AD patients, do not ameliorate the signs of dementia. By contrast, tau pathology, as mentioned above, seems to play a central role in the progression of the disease as the distribution of NFT, and not that of senile plaques, has a close temporal and spatial correlation with neuronal dysfunction and severity of dementia in AD patients [8,9]. In any case, despite the scientific progress that has been made in understanding the mechanism underlying AD, the interaction (crosstalk) and the relative contributions of Aβ and tau to cognitive deficits, synapse loss and neurodegeneration, still remains to be fully elucidated. 

Taking these considerations into account, the development of mouse models based on mutations in relevant genes such as amyloid precursor protein (APP) and presenilins 1 and 2 that participate in early-onset familial AD [10] should not be considered as “complete” AD-models. These amyloid models only partially reproduce AD pathology as they do not recapitulate aggregation of endogenous murine tau into neurofibrillary structures or the neuronal loss present in the human condition. Thus, they do not seem to be suitable to study the essential role of tau, as a mediator of amyloid toxicity, in the neurodegenerative process. On the other hand, since mutations in the gen *MAPT* are not found in AD patients, transgenic mice overexpressing human mutant tau, despite showing neuronal loss with age, could be considered models of tauopathies but not of AD [11].

Alternative methods have been applied to develop new AD animal models to move closer to what is happening in the patient and understand better the reciprocal in vivo interaction between Aβ and tau responsible of AD pathogenesis. For instance, Ribe et al. (2005) generated a double transgenic mouse (APP^sw^-tau^vlw^) obtained by crossing Tg2576 (expressing human APP with the double Swedish mutation) and VLW (expressing human 4-repeat tau containing a triple mutation, G272V, P301L and R406W) lines. They showed that, with age, the coexpression of human APP and Tau triggers neurofibrillary degeneration and neuronal loss in selective brain limbic areas [12]. Likewise, Oddo et al. (2003) generated the 3xTg-AD model with mutations in *APP*, *PS1* and *MAPT* [13]. This model exhibits plaques and synaptic dysfunction at 6-month-olds and develops tangle pathology at 12–15-months. Interestingly, in this model the clearance of Aβ decreases tau pathology, whereas augmenting tau levels does not modulate the onset or progression of amyloid pathology [14,15], suggesting that Aβ initiates the cascade of events leading to tau pathology. 

Viral vector-based systems, that through intracranial injections can be delivered in target brain areas, have been also used to study the progression and toxicity of tau [16]. The use of these vectors in different in vivo amyloid-models, makes possible to reproduce more accurately the events occurring in the human condition, offering the opportunity to obtain insight into the relationship between Aβ and tau and determine downstream key players of both pathways. Here, to study the interaction between amyloid and tau pathology, we injected an adeno-associated virus (AAV9) carrying the mutant P301L form of human tau, in the hippocampus of the APP/PS1 transgenic mouse model. This is an age-related model of amyloid deposition, mainly in the neocortex and the hippocampus [17], a region heavily affected in AD patients and with an important role in memory formation. After the AAV injections we assessed memory function using fear conditioning (FC) and Morris water maze (MWM) tests and we performed different biochemical analysis to evaluate the enhancement of the pathology, aggregation, and toxicity of human mutant tau in the presence of Aβ.

## 2. Results

### 2.1. Hippocampal Tau and Ptau Expression Using an AAV9-hTauP301L

**Figure 1:**
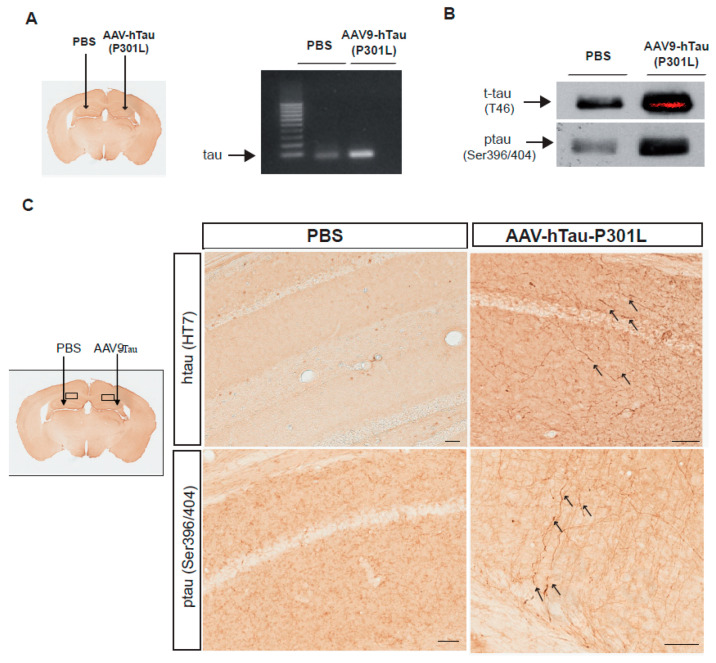
Overexpression of tau was detected in the AAV9-hP301LTau-injected hippocampus in comparison to PBS-injected side. Wild type (WT) mice were injected unilaterally with PBS or AAV9-hTauP301L in the hippocampus and analyzed 1 month later. (**A**) Polyacrylamide-gel analysis of the RT-PCR amplification of human tau transcript extracted from the hippocampus injected with PBS or with AAV9-hP301LTau. (**B**) Representative western blots of total tau (t-tau, using T46 antibody) and phospho-tau (ptau, Ser396/404, using PHF1 antibody) showing protein bands from hippocampus injected with PBS or AAV9-hTauP301LTau. (**C**) Representative images showing human tau and ptau (Ser396/404) immunoreactivity using HT7 and PHF1 antibodies in PBS- and in AAV9-hTauP301L- injected hippocampi (scale bar = 50 μm).

First, to verify the in vivo ability of the AAV9 viral vector to over express hTauP301L, a group of 6 wildtype (WT) mice received ipsilateral intra-hippocampal injections of AAV9-hTauP301L. One month later, three mice were sacrificed and hippocampi were dissected to analyze mRNA and protein expression levels of tau using T46 antibody, which recognizes both murine and human tau. An important overexpression of tau was detected both at mRNA and protein levels (Figure 1A,B) in the ipsilateral injected hippocampus in comparison to the contralateral non-injected hippocampus. Moreover, ptau levels were also detected using PHF1 antibody (which recognizes tau protein phosphorylated at serine residues 396 and 404) in the injected hippocampus vs control one, confirming the ability of AAV9-hTauP301L to induce tau phoshorylation in vivo. To analyze the presence and distribution of tau and ptau, one month after injection, three animals were perfused and their brains were analyzed by immunohistochemistry. HT7 antibody, which recognizes human total tau, and PHF1 antibody were used. A moderate axonal immunoreactivity for both antibodies, HT7 and PHF1, was shown in the AAV- injected hippocampus, suggesting that AAV9-hTauP301L can be used as a valuable tool to induce tau pathology in vivo. No significant human tau or ptau immunoreactivity was appreciated in the PBS-injected side (Figure 1C).

### 2.2. AAV9-hTauP301L Hippocampal Injections Accelerate Memory Deficits n APP/PS1 Mice 

**Figure 2:**
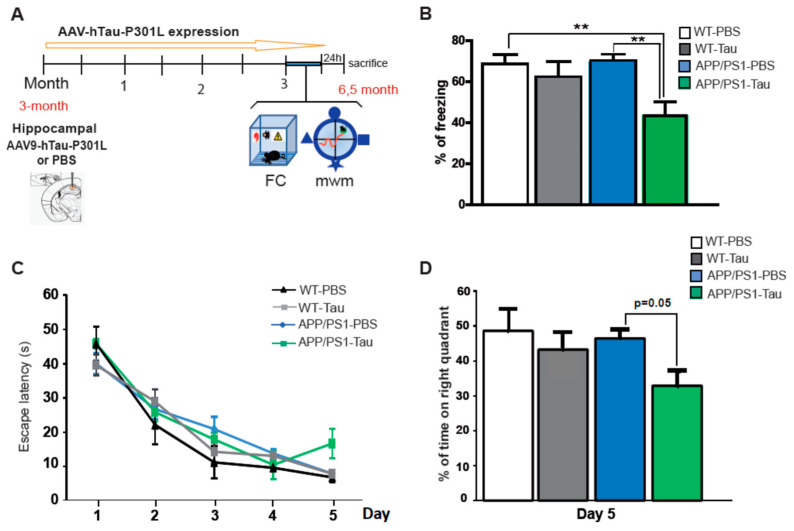
Wild type (WT) and APP/PS1 mice were injected with PBS or AAV9-hP301LTau in the hippocampus and analyzed 3 months later. (**A**) Scheme showing time-line for virus administration, behavioural tasks and sacrifice of mice. FC: fear conditioning, mwm: Morris water maze. (**B**) Freezing behaviour from WT and APP/PS1 mice treated with PBS or AAV9-hTauP301L. Data represent the percentage of time of freezing during the test. (**C**) Escape latency of the hidden-platform in the MWM test for the WT and APP/PS1 mice treated with PBS or AAV9-hTauP301L. (**D**) Percentage of time spent in correct quadrant during the probe test at Day 5. In all figures, results are expressed as mean ± SEM (*n* = 10–12 per group). Two-way analysis of variance (ANOVA) and Tukey’s post-hoc test was used for statistical analyses, ** *p* ≤ 0.01.

Since tau has been proposed as a mediator of Aβ-pathology in synaptic and memory function in AD [18], AAV9-hTauP301L was injected bilaterally in the dorsal hippocampi of 3-month-old APP/PS1 mice to analyze the interactions between tau and Aβ in the pathogenesis of AD. Likewise, AAV9-hTauP301L was also injected in 3-month-old WT mice. APP/PS1 and WT mice injected with PBS were used as control (*n* = 10–12 per group). Three months after viral injection, memory capacity was assessed using the fear-conditioning paradigm and the Morris water maze (MWM) test (timeline is shown in Figure 2A). First, mice were subjected to a contextual fear conditioning paradigm and tested 24 h later. A two-way ANOVA examined percentage of time spent freezing with genotype and treatment as factors (Figure 2B). Results revealed that there was a significant effect of treatment [F(1, 69) = 7.95, *p* < 0.01], without a significant effect of genotype [F(1, 69) = 2.12, *p* = 0.14] and no significant interaction of the two factors [F(1, 69) = 2.98.6, *p* = 0.08]. A Tukey’s post-hoc test revealed that AAV9-hTauP301L-APP/PS1 mice exhibited significant less freezing time than PBS-WT (*p* < 0.01) and PBS- APP/PS1 (*p* < 0.01) animals in the test session. These data indicate that a long-term overexpression of hTauP301L accelerates memory deficits in the fear conditioning test in APP/PS1 mice, but it does not affect fear memory in WT mice. To validate these results, learning and spatial memory was also assessed in these animals using the MWM task. In the spatial memory component of the test (hidden platform phase), no significant differences among groups where overall observed. In fact, the variance of the intra-group latencies in the hidden platform training (over trials) analyzed with the non-parametric Friedman test, demonstrated that the mean latency to reach the platform decreased as the training sessions progressed in every group. To study memory retention, mice were subjected to a probe trial in which the platform was removed 24 h after the last training session (Day 5). A two-way ANOVA examined percentage of time spent in the quadrant where the platform was located, with genotype and treatment as factors (Figure 2C). Results revealed that there was a significant effect of treatment [F (1, 39) = 5.65, *p* < 0.05], without a significant effect of genotype [F(1, 39) = 2.48, *p* = 0.12] and no significant interaction of the two factors [F(1, 69) = 1.05, *p* = 0.31]. The AAV9-hTauP301L-APP/PS1 group showed a poorer performance, with a time in right quadrant close to 25% (random), a Tukey’s post-hoc test revealed almost significant tendency (*p* = 0.05) between this group and PBS- APP/PS1 mice in the probe session (Figure 2D). These results support the idea that in the presence of amyloid, hTauP301L overexpression seems to accelerate the onset of memory deficits. 

### 2.3. Hippocampal AAV-9-hTauP301L Over-Expression in APP/PS1 Mice Accelerates Tau Pathology

**Figure 3:**
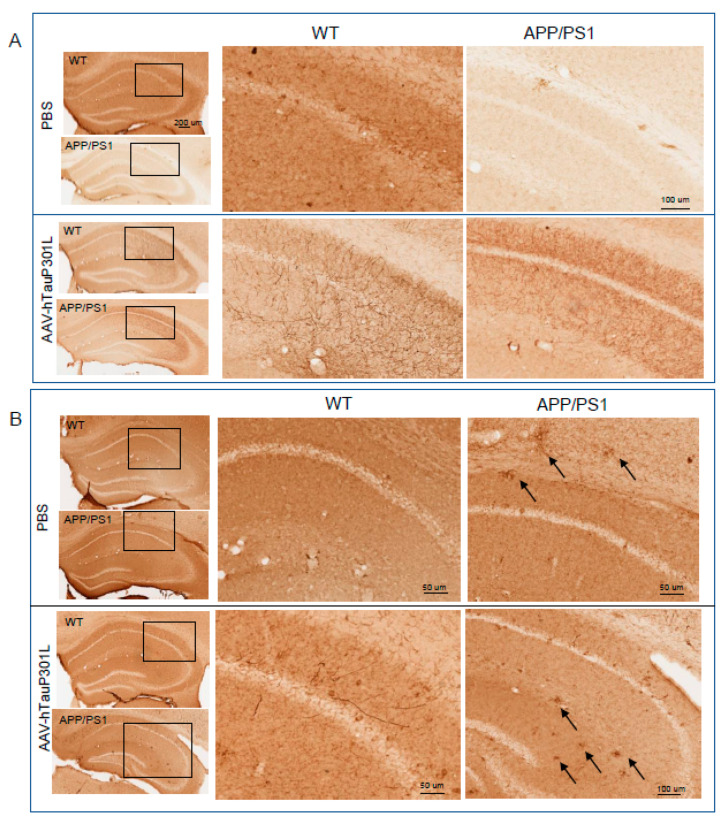
Hippocampal tau and ptau expression using an AAV9-hTauP301L. Wild type (WT) and APP/PS1 mice were injected with PBS or AAV9-hTauP301L in the hippocampus and analyzed 3 months later. (**A**) Representative images showing the expression of human tau (HT7 immunoreactivity) in WT and APP/PS1- AAV9-hTauP301L injected mice. No signal was observed in PBS-injected mice. Boxed areas of interest are shown in corresponding right panels. (**B**) Representative images showing ptau (Ser396/404) immunoreactivity using PHF1 antibody in PBS- and in AAV9-hTauP301L-injected mice. Boxed areas of interest are shown in corresponding right panels. In APP/PS1 mice, PHF1 immunoreactivity is evident in the vicinity of amyloid deposits (arrows).

To analyze the effectiveness of the viral vector employed for overexpressing hTauP301L, two to three animals per group were perfused to evaluate the immunoreactivity for tau and ptau. The human tau-specific monoclonal antibody HT7 was used to analyze the virus-derived tau expression in both WT and APP/PS1 injected mice. As depicted in Figure 3A (bottom panel), an abundant axonal immunoreactivity was observed in the hippocampi of both groups of AAV-hTauP301Linjected mice (WT and APP/PS1), with apparently no difference of expression levels between them. No significant human tau immnuoreactivity was appreciated at the same regions in PBS-injected mice (upper panels, Figure 3A). However, regarding ptau immunoreactivity (detected using PHF1 antibody, Figure 3B), it seems to be more abundant in the hippocampi of APP/PS1 than in WT PBS-injected mice (upper panels, Figure 3B). Moreover, an increase in ptau immunoreactivity (apparently associated to amyloid plaques) was observed in AAV-hTau-APP/PS1 mice when compare to PBS-APP/PS1 animals suggesting that the presence of hTau-P301L may also facilitate induction of tau pathology from endogenous *murine* tau. No significant HT7 or PHF1 immnuoreactivity was appreciated in WT animals or in other brain regions of AAV injected mice (data not shown), suggesting that the serotype used and/or the window of time expression employed, was not adequate to show a spreading of tau from hippocampus to other brain regions.

**Figure 4:**
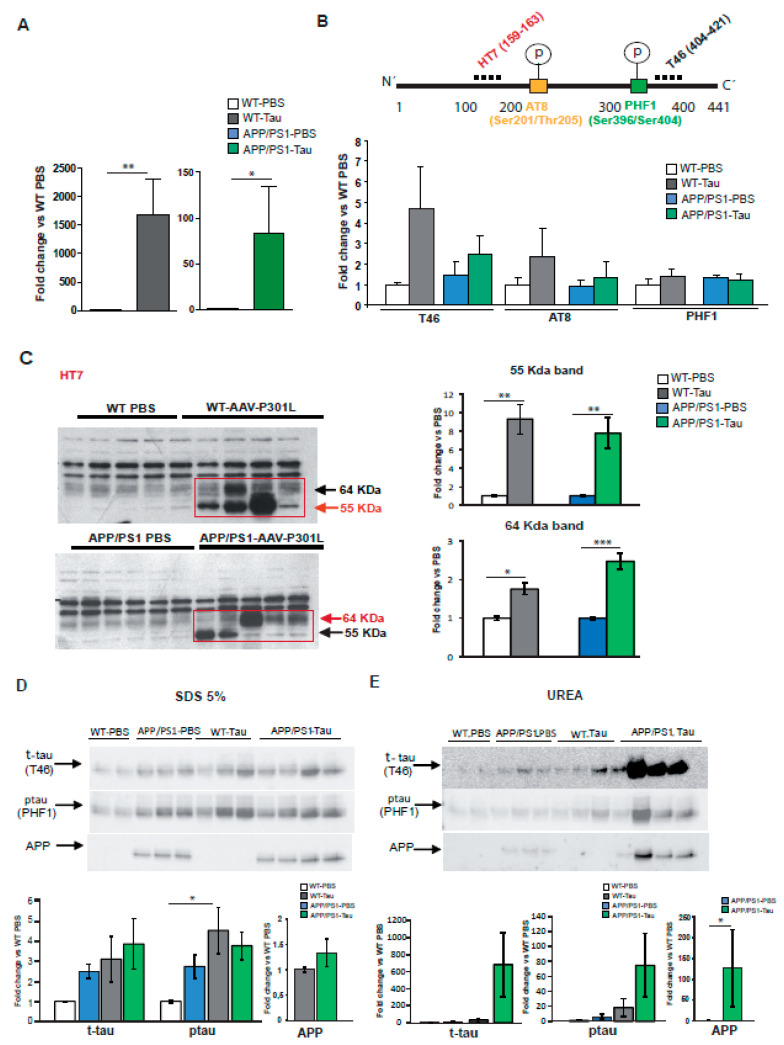
Hippocampal AAV-9-hTauP301L over-expression in APP/PS1 mice accelerates Tau pathology. (**A**) mRNA levels of human tau were detected by qRT-PCR in wild type (WT) and APP/PS1 mice injected with PBS or with AAV-9-hTauP301L (*n* = 3). (**B**) Schematic representation shows tau protein and the location of epitopes for the antibodies used in this study. Western blots showing the effects of PBS- or AAV9-hTauP301L injection in WT and APP/PS1 mice in tau and ptau expression using different antibodies to detect total tau (T46) and ptau (AT8, PHF1), normalized to actin, in 2% SDS hippocampal extracts (*n* = 5–6). (**C**) Same extracts were used to analyze the levels of human tau using HT7 antibody and bis/tris gels normalized to actin. Quantifications of the corresponding 55 and 64 Kda bands in the western blot are shown. Data are presented as mean ± SD; (*n* = 5–6). Statistics were performed by a Student’s *t* text. * *p* < 0.05, ** *p* < 0.01, *** *p* < 0.001 vs. PBS. (D-E) Western blots of 5% SDS-soluble fraction (S2, (**D**)) and insoluble fraction (urea, S3, (**E**)) probed for anti-total tau (T46), anti-ptau (PHF1) and anti-6E10 (recognize the amyloid precursor protein, APP) antibodies (*n* = 5–6). Statistics were performed by two-way ANOVA, * *p* < 0.05.

Next, to quantify specifically human tau (htau) levels, both qPCR (*n* = 3) and Western blot (*n* = 5–6) were employed. An important increase of mRNA htau levels was observed in the hippocampi of AAV-hTau injected mice when compared to their respective PBS-controls groups, in both WT (*p* < 0.01, Student *t* test) and APP/PS1 mice (*p* < 0.05, Student *t* test). To notice, the increase was more evident and significant in the case of WT than in the APP/PS1 group (Figure 4A). The analysis expression was followed up using total protein extracts (2%SDS) and specific antibodies to detect total, human and *murine*, tau (using T46 antibody) and phosphorylated forms of tau (using AT8 and PHF1 anitbodies). In this case, although an increase in total tau was observed in both WT- and APP/PS1- hTauP301L injected mice, no significant differences were encountered among the groups in these 2% SDS soluble extracts (Figure 4B). No important changes among groups were neither observed in phospho-tau species suggesting, that most of the pathological forms of tau might not be soluble in 2% SDS buffer [19]. 

Interestingly, a 64 kDa soluble form of tau (described as TBS-extracTable 64 kDa tau) involved in neurodegeneration has been described in the rTg4510 mouse model of tauopathy, and represents an early stage of tau assemby [20]. Thus, to further identify potential different soluble forms of tau, same extracts were separated using 4–12% Bis-Tris gels, transferred onto nitrocellulose membranes and incubated with the anti-human tau monoclonal antibody, HT7. Interestingly, although with a different pattern of expression between WT and APP/PS1 mice, both tau immunoreactive bands of 55–64 KDa were significantly increased in the hTauP301L injected groups compared to the PBS-injected mice (Figure 4C). Specifically, while a higher predominant expression of the 55 KDa band was observed in the WT-hTauP301L injected mice, the 64 KDa band, typically associated with a more pathological form [19], was more predominant in the case of APP/PS1-hTauP301L injected mice. This result is in line with our immunohistochemical observations and reinforces the idea that the presence of amyloid facilitates tau pathology. The presence of this different tau expression pattern may also explain why the overexpression of hTauP301L seemed to disrupt memory function in APP/PS1 mice but did not affect that of WT animals.

Next, in order to investigate likely insoluble forms of tau, the other hippocampus was used to obtain solubility fractionation of granular tau oligomers [21]. Similar to the results found in 2% SDS extracts, in a first TBS soluble fraction, despite inter-animal differences within hTauP301L-injected mice, an increase of soluble total tau was found in injected animals (data not shown). Next, considering that tau pathology is usually associated with a transition of tau from soluble to insoluble forms, 5% SDS and urea extracts were used to analyze differential solubility of tau species. In this fraction, total tau levels (T46) were similarly increased in APP/PS1-PBS and in WT and APP/PS1- hTauP301L injected mice groups (Figure 4D), however, in the insoluble fraction (urea), although not significant, an evident accumulation of total insoluble tau was mainly observed in the case of hTaup301L-APP/PS1 mice (Figure 4E). In the same line, these animals showed an increase of phospho-tau (PHF1) levels in the urea fraction while in the 5% SDS fraction, the APP/PS1-PBS mice showed similar levels to injected animals (Figure 4D). These results let us hypothesize that the accelerate phenotype observed in hTaup301L-APP/PS1, may be induced, at least in part, by the presence of these tau aggregates.

Importantly, since no tau was detected in the urea fraction in hP301L-tau WT injected mice (Figure 4E), it seems that the presence of amyloid is necessary to induce tau aggregation and that Aβ and tau may act synergistically to accelerate the formation of insoluble forms of hyperphosphorylated tau. In line with this idea, to determine whether tau could also affect or accelerate amyloid pathology, in the same extracts hAPP levels were analyzed. Interestingly, while similar levels of hAPP were observed between hTauP301L-APP/PS1 and PBS-APP/PS1 mice in the 5% SDS fraction (Figure 4D), in the urea fraction, an important accumulation of hAPP was specifically observed in the hTau-P301L-APP/PS1 mice (*p* < 0.05; Figure 4E). This specific accumulation indicates that in the presence of hTauP301L, there is also a transition of APP from soluble to insoluble form. Taken together, it seems that tau and amyloid reciprocally alter each other accumulation and that the convergence of amyloid and tau pathology may subsequently accelerate cognitive decline.

### 2.4. Amyloid Mediates the Accumulation of Tau in Dendritic Mitochondria 

**Figure 5:**
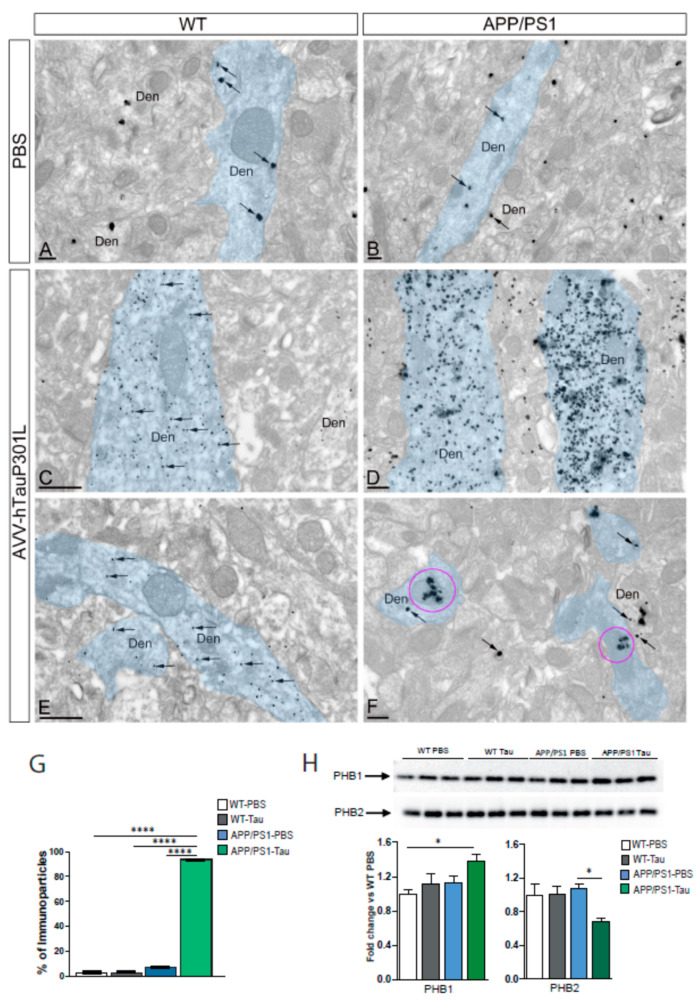
Electron micrographs showing the distribution of immunoparticles for tau in the CA1 region of mice hippocampi using a pre-embedding immunogold technique. (**A**,**B**) In WT and APP/PS1 in control group (PBS), few immunoparticles for tau (arrows) were observed in the cytoplasm of dendritic shafts (Den) of pyramidal cells (pseudo coloured in blue). Immunoparticles for tau were not associated with mitochondria present in the shafts. (**C**,**D**) In WT and APP/PS1, in AVV-hTauP301L group, many immunoparticles for tau (arrows) were observed in the cytoplasm of dendritic shafts (Den) of pyramidal cells (pseudo coloured in blue). However, only in APP/PS1, immunoparticles were mostly associated with mitochondria (purple circle, (**F**)) but not in WT mice, where the mitochondria remain free of tau immunoparticles (**E**). Scale bars: (**A**,**B**,**D**,**F**): 200 nm; (**C**,**D**): 500 nm. (**G**) Histogram showing the quantification of tau immunoparticles in the dendritic mitochondria for each group of animals (*n* = 3 animals/group). Error bars indicate SEM; *p* < 0.0001. (**H**) Hippocampal PHB1 and PHB2 levels assayed by immunoblotting in WT, and APP/PS1 mice injected with the AAV9-hTauP301L (*n* = 4–5). Equal loading of the gels was assessed by Ponceau staining. Two-way ANOVA and Tukey’s post-hoc test was used for statistical analyses. * *p* < 0.05, **** *p* < 0.0001.

One of the mechanisms proposed regarding the increase of amyloid toxicity in the presence of tau is that amyloid increases the mislocalization of tau to the somato-dendritic compartment in AD mouse models [10,22]. Thus, next we analyzed the subcellular distribution of tau immunoparticles in the CA1 region of all the experimental groups (*n* = 3 animals per group) using a pre-embedding immunogold technique with the T46 antibody. As depicted in Figure 5, a diffuse distribution and few number of T46 conjugated gold particles was observed mostly within the dendrites in PBS-injected mice, both WT (Figure 5A) and APP/PS1 mice (Figure 5B). An evident and marked increase in the number of tau particles within the dendrites was observed in both WT (Figure 5C) and APP/PS1 (Figure 5D) mice injected with the virus. Interestingly, WT mice expressing TauP310L reveal sparse gold particles (arrows, Figure 5C) in contrast to numerous large clusters observed in the dendrites of APP/PS1 mice expressing TauP310L (arrows, Figure 5D). This is in concordance with the presence of higher molecular weight band (64 kDa) observed in Figure 4C and the higher levels of total tau in the urea-insoluble fraction (Figure 4E) observed in the APP/PS1-hTauP301L group of mice. These data confirmed previous observations indicating that the presence of amyloid induces tau aggregation in dendrites leading to the loss of dendritic spines and therefore accelerating the onset of cognitive deficits [22,23,24].

In this line, it has been described that the presence of dendritic tau induced by Aβ_42_ alters the transport of vesicles and organelles such as the mitochondria [25,26]. As depicted in Figure 5F, the presence of tau inmunoparticles in the mitochondria is very evident in the APP/PS1-hTauP301L, whereas most of the mitochondria in the WT- hTauP301L group remain free of tau inmunoparticles (Figure 5E), despite the increase in tau immunoreactivity observed in the dendritic shafts (Den). Quantitative analysis of pre-embedding gold particles in the mitochondria present along dendritic shafts, either associated with the membrane surface or with intracellular sites, showed clear differences among the groups, revealing an aberrant accumulation of tau in the mitochondria in the case of APP/PS1 mice injected with the AAV-hTauP301L compared to the other groups (93.70% in APP/PS1-tau, *n* = 559 particles, and 7.20% in WT-tau, *n* = 399 particles; 2.95% in APP/PS1-PBS, *n* = 299 particles, and 2.70% in WT-PBS, *n* = 295 particles), (two-way ANOVA test and Tukey’s post hoc test, *p* < 0.0001; Figure 5G). No differences were observed between WT and APP/PS1 treated with PBS. 

One of the molecules localized in the inner mitochondria with a possible direct link between mitochondrial dysfunction and tau pathology and AD is the prohibitin (PHB) complex, composed of PHB1 and PHB2 subunits [27,28]. Thus, we analyzed whether the protein expression of PHB1 and PHB2 is altered in these animals (Figure 5H). Results revealed that there was a significant effect of treatment in PHB1 expression [F(1, 8) = 4.86, *p* = 0.05], and a significant effect of genotype [F(1, 8) = 5.86, *p* < 0.05] and no significant interaction of the two factors [F(1, 8) = 0.617, *p* = 0.45]. A Tukey’s post-hoc test revealed a significant increase (+38%) between the APP/PS1-hTauP301L group and the WT-PBS (*p* < 0.05). Regarding PHB2, results revealed significant effect of treatment [F(1, 8) = 4.87, *p* = 0.05], a non-significant effect of genotype [F(1, 8) = 2.15, *p* = 0.18] and a significant interaction of the two factors [F(1, 8) = 5.21, *p* = 0.05]. A Tukey’s post-hoc test revealed a significant decrease (−32%) between the APP/PS1-hTauP301L group and the APP/PS1-PBS (*p* < 0.05). The reverse protein profile observed in APP/PS1-tau mice for both PHB subunits could point out not only a mitochondrial dyshomeostasis but also an imbalance in survival/apoptotic mechanisms, due to both subunits has the possibility to be located also at plasma membrane (interacting with pivotal signalling routes) and at nuclear level (transcriptional modulators) [29].

## 3. Discussion

In this study an adeno-associated virus (AAV) carrying the mutant P301L form of human tau under the synapsin promoter was injected in the hippocampus of the APP/PS1 transgenic mouse model to study the possible crosstalk between amyloid and tau in the development of AD. Our data indicated that the overexpression of hTauP301L accelerates memory deficits in the APP/PS1 mice. Interestingly, the overexpression of hTauP301L does not affect memory in WT mice, suggesting that amyloid can potentiate tau aggregation and phosphorylation and confirming the gain of toxic function hypothesis for the effect of amyloid on tau. 

In agreement with our studies, it has been consistently shown that amyloid induces tau pathology in different animal models, whereas tau does not aggravate amyloid toxicity. Likely, in AD patients, Aβ accelerates subcortical tau-pathology, suggesting that is probably Aβ accumulation that triggers, aggravates or accelerates the pathology of tau (review in [22,30]). Accordingly, by crossing the APP/PS1 mouse with a model expressing wild type human tau (rTg21221), authors showed an increase in amyloid deposits and dystrophic neurite number that did not aggravate Aβ-mediated synapse loss [31]. By contrast, other authors consider that tau cannot be considered as a second player and both pathologies coexist, and work together in a synergistic manner [32,33]. Likewise, as an alternative hypothesis different studies suggest that other factors, Aβ-independent, contribute to the progression of tau pathology in AD [5]. Nowadays, the respective contribution of each pathology, the chronology and their interaction in AD development is still a matter of debate. 

Our data clearly show a transition from soluble to insoluble forms of tau in the APP/PS1 mice injected with the AAV-hTauP301L in comparison with the WT-injected animals. The aggregation of tau takes place both at extracellular and intracellular levels. It is known that APP/PS1 mice exhibit ptau neuritic processes nearby the amyloid deposits [17], and interestingly, we observed higher ptau immunoractivity around the characteristic deposits in APP/PS1-hTau mice than in APP/PS1 control mice (Figure 3B). Since the deposits were reactive to PHF1 but not to HT7 antibody (Figure 3A), we hypothesized that is the endogenous wild-type murine tau that is being recruited in the vicinity of the amyloid deposits. Similar results were previously reported in a detailed analysis of an APP/PS1/tau model (5xFAD × Tg30 mice), in which they detected higher levels of insoluble endogenous murine tau in the brains of 9-month-old 5xFAD × Tg30 mice, but not in Tg30 (mutant human tau transgenic mice) [34]. These data indicate that murine tau has the ability to aggregate but, since APP/PS1 models are not able to recruit endogenous murine tau into NFT, even in the presence of wild-type human tau [35], is evident that in the presence of amyloid, some particles of human mutant tau are needed for “seeding” and propagating the disease.

We also detected a marked increase in the number of gold tau particles (T46 antibody) within the dendrites in both WT (Figure 4C) and APP/PS1 (Figure 4D) mice injected with the virus. To notice, in the APP/PS1 mice the particles formed large clusters in comparison to the sparse particles observed in WT mice. In particular, we found an accumulation of tau particles in the dendritic mitochondria in APP/PS1-tau that can lead to mitochondrial dysfunction and prevent neurons from functioning normally. The presence of tau in the dendrites may represent an early event preceding dendritic spines loss, local increase in Ca^2+^, microtubule destabilization and mitochondria depletion [25]. It has been previously shown that the overexpression of tau in hippocampal neurons leads to misslocalization of tau into the somatodendritic compartment and affects the transport of mitochondria and organelles triggering the degeneration of synapses due to local energy deficiency in the dendrites [26,36].

Interestingly, the localization of some forms of tau in the mitochondria is an early pathological event that can be a trigger of the neuronal dysfunction in tauopathies [37]. Specifically, in the case of AD, mitochondrial abnormalities due to axonal trafficking impairments caused by destabilization of microtubules mediated by hyperphosphorylated tau is an early event that contribute to the development of the disease [38]. In line with this idea, several authors have suggested that mitochondrial PHB complex alterations, that seems to be specifically disrupted in our hTauP301L-APP/PS1 injected mice (Figure 5), are associated with neuroplasticity deficits [39] and more specifically, with tau phosphorylation and neurodegeneration [28]. The PHB complex has been reported to show altered patterns of expression in different neurodegenerative diseases such as Parkinson’s disease (PD), amyotrophic lateral sclerosis (ALS) and frontotemporal dementia [40,41]. 

It is also possible that soluble forms of tau alter the distribution of mitochondria throughout the neuron, leaving the synapses deprived of their high energy requirements [42], becoming more vulnerable to amyloid or any insult and resulting in synaptic dysfunction. Thus, it is evident that the synapse degeneration and memory loss in neurodegenerative disorders arises in part from mitochondrial dysfunction [43]. Accordingly, several studies support the idea that tau may have a synergistic effect on Aβ-induced mitochondrial dysfunction [44]. One of the hypothesis is that different forms of tau affects mitochondrial membrane potential and increases the mitochondrial sensibility to different stressors, such as Aβ and calcium [45,46,47]. 

Overall, our data demonstrated that mitochondrial dysfunction may contribute to the memory impairment generated in the APP/PS1 mouse model by overexpressing the human mutant form of tau, TauP301. The mitochondrial dysfunction is mediated by the accumulation of anomalous tau in the mitochondria, which takes place in the presence of amyloid in the APP/PS1 model. Our results represent a novel mechanism by which amyloid and tau may contribute to the pathogenesis of AD. It would be great to study deeper the effect of tau on Aβ-induced impaired mitochondrial functioning and try to see if the restoration of these mitochondrial alterations could help improving AD pathophysiology.

## 4. Materials and Methods

### 4.1. Animals and Stereotaxic Injection

Recombinant AAV vector of serotype 9 were produced as described in [48] ] to express human mutant TauP301L in the longest tau isoform (Tau4R/2N) under control of the human synapsin1 gene promoter using the plasmid donated by Dr. Sebastian Kügler [49].

Two months-old wild-type (WT) C57BL/6 mice were used to test the transfection capacity of the AAV9-hTau-P301L. Three-four months old, male and female, APP/PS1 mice and their negative littermates were used to test the toxicity of the AAV9-hTau-P301L in the presence of Aβ. The APP/PS1 model is on an inbred C57BL/6J genetic background, expresses human transgenes for the APP bearing the Swedish mutation and for the PSEN1 containing a L166P mutation, both driven by the Thy1 promoter [17]. Intracerebral injections of viral particles were performed stereotactically into the dorsal hippocampus (AP − 2 mm, ML ± 1.4 mm, DV − 1.7 mm relative to bregma) as described in [48]. Standard injection was 1 µL of a viral suspension containing 10^E9^ transducing units using 5 µL glass syringes with a fixed needle (Hamilton, Reno, Nevada). After injection at a rate of 0.25 µL/min, the needle was left in place for 5 min before withdrawal.

Animals were housed 4–6 per cage with free access to food and water, and maintained in a temperature-controlled environment on a 12 h light–dark cycle. All procedures were carried out in accordance with the current European and Spanish regulations (2010/63/EU; RD52/2013) and the study was approved by the Ethical Committee of the University of Navarra.

### 4.2. Behavioral Studies

All behavioral studies used in this study were carried out during light time, from 9:00 to 16:00 h. Fear conditioning and Morris water maze paradigms were used to analyze the effect of AAV9-hTau-P301L on fear memory of APP/PS1 mice.

#### 4.2.1. Fear Conditioning Test (FC)

The task was carried out in a StartFear system (Panlab) as described in [48]. Briefly, on Day 1, mice were habituated to the conditioning chamber for 3 min with no stimuli presented. After twenty-four hours mice were placed in the same chamber and were allowed to explore for 2 min. After that, they received two footshocks (0.3 mA) of 2 s separated by an interval of 30 s and were returned to their home cages after another 30 s. The following day, mice were returned to the conditioning chamber and allowed to explore the context for 2 min. Freezing behavior was recorded during this time and freezing scores were expressed as percentages. 

#### 4.2.2. Morris Water Maze Test (MWM)

The task was carried out in a StartFear system (Panlab) as described in [48]. Briefly, during the visible-platform phase were trained to find a platform raised above the surface of the water (and no visible cues) during three consecutive days and eight trials per day. Next, animals undertook the hidden-platform phase, in which mice were trained during 5 consecutive days (four trials per day) to locate a platform submerged 1 cm beneath the water surface with the help of some visible cues present in the walls of the swimming pool. On Day 6, mice were subjected to a probe trial in which they swam for 60 s in the pool without the platform. All trials were monitored by a camera connected to a SMART-LD program (Panlab) for subsequent analysis of escape latencies during visible and hidden platform phases and percentage of time spent in each quadrant of the pool during the probe trial. All experimental procedures were performed blind to groups. 

### 4.3. Quantitative Real-Time PCR

Total RNA was isolated from the correspondent mice or human tissue using Trizol reagent (Sigma-Aldrich, St. Louis, MO, USA). The RNA was treated with DNase at 37 °C for 30 min and reverse-transcribed into cDNA using SuperScript^®^ III Reverse Transcriptase (Invitrogen, Waltham, MA, USA). Quantitative real-time PCR was performed to quantified gene expression. Assays were undertaken using a Power SYBR Green PCR Master Mix (Applied Biosystems, Waltham, MA, USA) and the corresponding specific primers: human Tau/Fw: 5′-ATGGAAGATCACGCTGGGAC-3′ Rev: 5′-TGGTCTTGGTGCATGGTGTAG-3′; 36B4/Fw: 5′-AACATCTCCCCCTTCTCCTT-3′ Rev: 5′-GAAGGCCTTGACCTTTTCAG-3′). Real-time was carried out using an ABI Prism 7300 sequence detector (Applied Biosystems, Waltham, MA, USA) and data were analyzed using the Sequence Detection Software v.3.0 (Applied Biosystems, Waltham, MA, USA). Relative gene expression was calculated in reference to the control group using the DDCT method [50].

### 4.4. Protein Extracts and Fractionated Brain Lysates

To obtain total protein extracts, half mice hippocampi were homogenized in lysis buffer with protease inhibitors (10 mM Tris-HCl ph = 7.5, 1 mM NaF, 0.1 mM Na_3_VO_4_, 2% SDS), sonicated for 2 min, left 20 min on ice and centrifuged at 13,000 rpm for 13 min at 8 °C. The supernatant was stored at −80 °C. Total protein concentrations were determined using the Pierce^TM^ BCA Protein Assay kit (Thermo Scientific, Waltham, MA, USA). The other half mice hippocampi were used to obtain fractionation of granular tau oligomers [21]. Tissues were homogenized in aqueos buffer (50 mM Tris-HCl pH 7.4, 175 mM NaCl, 2% SDS) and centrifuged first at 1000 g for 2 min to remove unhomogenized debris and then for 2 h at 200,000× *g* (4 °C). The pellet (P1) was resuspended in 5% SDS buffer (50 mM Tris-HCl pH 7.4, 175 mM NaCl, 5% SDS) and centrifuged for 2 h at 200,000× *g* (25 °C). S2 is the detergent-soluble fraction. P2 was resuspended in buffer containing 8 M urea (50 mM Tris-HCl pH 7.4, 175 mM NaCl, 8% SDS, 8 M urea) with agitation for 18 h at room temperature. S3 is the detergent-insoluble fraction. Except in S3, protein content was determined using Thermo Scientific™ Pierce™ BCA™ Protein Assay (Bio-Rad, Hercules, CA, USA).

### 4.5. Immunoblotting 

Thermo Scientific™ Pierce™ BCA™ Protein Assay (Bio-Rad, Hercules, CA, USA). samples (15–20 μg) were mixed with 6× Laemmli sample buffer, boiled at 95 °C for 5 min, resolved onto SDS-polyacrylamide gels and transferred to nitrocellulose membrane. In addition, we use bis-tris polyacrylamide gels [51] to obtain a higher band resolution since these gels have a neutral pH that minimizes protein modifications. 

Membranes were then blocked for 1 h with 5% milk in TBS and incubated overnight with the corresponding primary antibody: mouse monoclonal anti-htau (HT7, 1:1000, Invitrogen), mouse monoclonal anti-ptau Ser202:Thr205 (AT8, 1:1000, Pierce), mouse monoclonal anti-tau (T46, 1:1000, Sigma-Aldrich, St. Louis, MO, USA), mouse monoclonal anti-β-actin (1:100,000, Sigma-Aldrich, St. Louis, MO, USA), rabbit polyclonal anti-Prohibitin 1 (Phb1), and anti-Prohibitin 2 (Phb2), (1:1000, Cell Signalling, Woburn, MA, USA). 

Following two washes in TBS/Tween-20 and one in TBS alone, immunolabeled protein bands were detected with an HRP-conjugated anti-mouse antibody (1:5000, Santa Cruz, Dallas, TX, USA). Antibody binding was then visualized by enhanced chemiluminescence system (ECL, GE Healthcare Bioscience, Buckinghamshire, UK), and autoradiographic exposure to ChemiDoc™ Imaging System (Bio-Rad, Hercules, CA, USA). Finally, proteins were quantified based on the optic density of the band and normalized using β-actin as load control, thanks to the Image Lab™ Software 6.0.1. 

### 4.6. Immunohistochemistry

Under xylazine/ketamine anesthesia, animals from both groups (*n* = 4) were perfused transcardially with saline and 4% paraformaldehyde in phosphate buffer (PB). 

Immunohistochemical analysis was conducted on 40 µm hippocampal sections, derived from animals perfused transcardially with saline and 4% paraformaldehyde in phosphate buffer (PB) to detect human tau expression using mouse monoclonal antibody HT7 (Invitrogen, diluted 1:500) or mouse monoclonal antibody PHF1 (gift of Peter Davis, diluted 1:1000) following the protocol previously described [52]. The technique was optimized by mainly incorporating two additional steps: a 30-min unmasking-step in 0.01 M citrate buffer pH 6 at 60 °C prior to quenching of endogenous peroxidase, and a 1 h blocking-step in PBS-Triton 0.2% and 5% normal serum (same source as for the secondary antibody) at RT before incubation with primary antibody. The former allowed the retrieval of antigen (since proteins typically crosslink during fixation), while the latter prevented non-specific binding of antibodies to tissue or Fc receptors.

### 4.7. Pre-Embedding Immunogold Electron Microscopy

Immunohistochemistry for electron microscopy was performed using the pre-embedding immunogold technique as previously described [53]. Mice were deeply anesthetized and perfused with 4% paraformaldehyde and 0.05% glutaraldehyde in 0.1 M phosphate buffer (PB, pH = 7.4). Brains were then immersed in the same fixative for 2 h and they were processed in 60 μm-thick coronal sections using a vibratome. Sections were incubated with 3–5 μg/mL of the primary antibody, mouse monoclonal anti-tau antibody, clone tau46, (Catalogue Number T9450, Sigma-Aldrich), in TBS containing 1% NGS for 24 h at 4 °C. After several washes in TBS, sections were incubated for 2 h in goat anti-rabbit IgG coupled to 1.4 nm gold (Nanoprobes Inc., Stony Brook, NY, USA) diluted 1:100 in TBS containing 1% NGS. Sections were then postfixed in 1% glutaraldehyde dissolved in PBS. They were washed in double distilled water, followed by silver intensification through an HQ silver kit (Nanoprobes Inc., Stony Brook, NY, USA). The labelled sections were then treated with OsO_4_ (1% in 0.1 M PB), block-stained with 1% uranyl acetate, dehydrated in a graded series of ethanol and flat-embedded on glass slides in Durcupan resin (Sigma-Aldrich, St. Louis, MO, USA). Regions of interest were cut at 70–90 nm on an ultramicrotome (Reichert Ultracut E, Leica, Vienna, Austria) and collected on single slot pioloform-coated copper grids. Staining was performed on drops of 1% aqueous uranyl acetate followed by Reynold’s lead citrate. Ultrastructural analyses were performed in a JEOL-1400 Flash electron microscope (Jeol Ltd., Tokyo, Japan).

### 4.8. Quantification and Analysis of Pre-Embedding Immunogold Data

To establish the possible association of tau immunoreactivity in mitochondria compartments of pyramidal cells between WT and APP/PS1 PBS and htauP301L injected mice, we used 60-μm-thick coronal slices processed for pre-embedding immunogold immunohistochemistry. The procedure was similar to that used previously [53]. Briefly, three samples of tissue were obtained for the preparation of embedding blocks. To minimise false negatives, electron microscopic serial ultrathin sections were cut close to the surface of each block, as immunoreactivity decreased with depth. We estimated the quality of tau immunolabelling by always selecting areas with optimal gold labelling at approximately the same distance from the cutting surface. Randomly selected areas were then photographed from the selected ultrathin sections and used with final magnification between 30,000 and 50,000×. We counted immunoparticles identified in each reference area and associated with mitochondria that are present along dendrites. The data were expressed as a percentage of immunoparticles for tau un mitochondria, either associated with the membrane surface or with intracellular sites.

### 4.9. Data and Statistical Analyses

The results were processed for statistical analysis using GraphPad PRISM, version 5.03. Unless otherwise indicated, results are presented as mean ± standard error of the mean (SEM). The normal distribution of the data was checked by the Shapiro–Wilk test. Two-way analysis of variance (ANOVA) and Tukey’s post-hoc test was used for statistical analyses of data. In the MWM test, latencies to find the platform were analyzed by two-way repeated measures ANOVA test (genotype x trial) followed by the Bonferroni’s *post hoc* test to compare cognitive status among groups. An unpaired, two-tailed Student’s *t*-test was used to compare two groups. Statistical significance was set at * *p* ≤ 0.05 or ** *p* ≤ 0.01.
